# The Aetiologies and Impact of Fever in Pregnant Inpatients in Vientiane, Laos

**DOI:** 10.1371/journal.pntd.0004577

**Published:** 2016-04-06

**Authors:** Vilada Chansamouth, Syvilay Thammasack, Rattanaphone Phetsouvanh, Valy Keoluangkot, Catrin E. Moore, Stuart D. Blacksell, Josee Castonguay-Vanier, Audrey Dubot-Pérès, Jarasporn Tangkhabuanbutra, Narongchai Tongyoo, Phooksavanh Souphaphonh, Onanong Sengvilaipaseuth, Manivanh Vongsouvath, Koukeo Phommasone, Davanh Sengdethka, Amphayvanh Seurbsanith, Scott B. Craig, Laura Hermann, Michel Strobel, Paul N. Newton

**Affiliations:** 1 Lao-Oxford-Mahosot Hospital-Wellcome Trust Research Unit (LOMWRU), Microbiology Laboratory, Mahosot Hospital, Vientiane, Lao PDR; 2 Institut de la Francophonie pour la Medecine Tropicale (IFMT), Vientiane, Lao PDR; 3 Infectious Disease Center, Mahosot Hospital, Vientiane, Lao PDR; 4 Centre for Tropical Medicine and Global Health, Nuffield Department of Medicine Research Building, University of Oxford, United Kingdom; 5 Mahidol–Oxford Research Unit (MORU), Faculty of Tropical Medicine, Mahidol University, Bangkok, Thailand; 6 UMR_D 190 “Emergence des Pathologies Virales”, Aix- Marseille University, IRD French Institute of Research for Development, EHESP French School of Public Health, Marseille, France; 7 Department of Gynecology and Obstetrics, Mahosot Hospital, Vientiane, Lao PDR; 8 WHO/FAO/OIE Collaborating Centre for Reference and Research on Leptospirosis, Queensland Health Forensic and Scientific Services, Australia; 9 Department of Virology, Armed Forces Research Institute of Medical Sciences (AFRIMS), Bangkok, Thailand; Faculté de Médecine,Aix-Marseille Université, FRANCE

## Abstract

**Introduction:**

Laos has the highest maternal mortality ratio in mainland Southeast Asia and a high incidence of infectious diseases. Globally, malaria has been the pathogen most intensively investigated in relation to impact on pregnancy, but there has been relatively little research on the aetiology and impact of other diseases. We therefore aimed to determine the causes and impact of fever in pregnant women admitted to two central hospitals in Vientiane City, Lao PDR (Laos).

**Materials and Methods:**

This hospital-based prospective study was conducted in Mahosot Hospital and the Mother and Child Hospital, Vientiane, between 2006 and 2010, with the aim to recruit 250 consenting pregnant women admitted with tympanic temperature ≥37.5°C. Primary outcome was the cause of fever and secondary outcomes were pregnancy outcomes. Specific investigations (culture, antigen, molecular and serological tests) were performed to investigate causes of fever. After discharge, all pregnant women were asked to return for review and convalescence serum on day 10–14 and were monitored until delivery.

**Principle findings:**

250 pregnant women were recruited to this study between February 2006 and November 2010. Fifty percent were pregnant for the first time. Their median (range) gestational age on admission was 24 (4–43) weeks. The median (range) tympanic admission temperature was 38.5°C (37.5–40.5°C). Fifteen percent of patients stated that they had taken antibiotics before admission. Headache, myalgia, back pain and arthralgia were described by >60% of patients and 149 (60%) were given a laboratory diagnosis. Of those with confirmed diagnoses, 132 (53%) had a single disease and 17 (7%) had apparent mixed diseases. Among those who had a single disease, dengue fever was the most common diagnosis, followed by pyelonephritis, scrub typhus, murine typhus and typhoid. Patients were also diagnosed with tuberculosis, appendicitis, *Staphylococcus aureus* septicemia, leptospirosis, Japanese encephalitis virus infection and *Plasmodium falciparum* malaria. Severe consequences, including maternal death, miscarriage, stillbirth, low birth weight and preterm birth, were found among 28 (78%) mothers with dengue fever, rickettsioses and typhoid.

**Conclusion:**

Fevers other than malaria, such as dengue, pyelonephritis, rickettsioses and typhoid are common causes of fever during pregnancy in the Asian tropics. Further investigations of their impact in the community on maternal death, fetal loss, vertical transmission, low birth weight and preterm birth are needed.

## Author Summary

Laos has a high incidence of infectious diseases and high prevalence of poverty. Malaria in pregnancy if of great public health interest. However, malaria has not been reported in Vientiane City for decades, but others diseases such as dengue fever, rickettsioses, typhoid fever, leptospirosis and Japanese encephalitis, have. In this prospective hospital-based study of the causes of fever in pregnancy, dengue fever was the most common diagnosis, followed by pyelonephritis with *Escherichia coli*, murine typhus, scrub typhus and typhoid fever. Severe consequences, including maternal death, miscarriage, stillbirth, low birth weight and preterm birth, were found among mothers with dengue fever, rickettsioses and typhoid. As a hospital-based study it has limitations and large community cohort studies will help us to learn more about fevers and their impact in pregnancy.

## Introduction

Maternal death and poor birth outcomes remain significant public health problems in much of the world [1,2]. Most of the mortality and morbidity burden around delivery are related to direct obstetrical problems such as bleeding. However, there is relatively little information on the importance of the great diversity of infectious diseases on pregnancy outcome, especially in the tropics.

Some infectious diseases are more common or more severe in pregnancy (e.g. *Plasmodium falciparum*, *Listeria monocytogenes*, hepatitis E virus (HEV), herpes simplex virus and influenza) [3–9]. However, for most common causes of fever in the tropics there is little evidence as to whether pregnant women are at higher risk of disease, of severe disease and what the impacts are on mothers and their offspring, by pathogens and gestational age [10–14]. Understanding of the relationship is impaired as deaths from infectious diseases and deaths in pregnancy are often not connected in public health surveillance.

Anatomical and physiological changes may predispose pregnant women to certain infections, such as those of the urinary tract. These occur in ~8% of pregnant women, causing pyelonephritis in 1–2%, most commonly during the second trimester, and may lead to preterm birth and low birth weight [15–18]. Listeriosis is reported to be 20 times more common during pregnancy than in the non-pregnant population, likely because of immunosuppression during pregnancy [4] with chorioamnionitis, placental abscesses, abortion, prematurity, fetal distress, vertical transmission and neonatal death reported [19]. Adverse birth outcomes such as maternal death, premature delivery, and low birth weight have been well documented in pregnant women with *P*. *falciparum* and *P*. *vivax* infection [20–22]. Dengue, leptospirosis, typhoid fever and rickettsioses are common causes of fever in tropical areas [23,24] but whether the risk and severity of these diseases are higher in pregnancy is unclear [17, 25–36].

The Lao PDR (Laos) is a tropical country in Southeast Asia with a high burden of infectious diseases. Although, Laos achieved 75% reductions in maternal mortality ratio (MMR) from 1990–2015, it still has the highest estimated MMR in mainland Southeast Asia (197/100,000 live births) [1,2]. Direct causes such as haemorrhage and eclampsia are the most common reasons [37]. Although some indirect causes of maternal death, such as malaria, have received global focus, other fevers have been neglected and there is a meager evidence base. We aimed to determine the causes and impact of fever in pregnant women admitted to two central hospitals in Vientiane City, Laos, to provide evidence on the aetiology and management of fever in pregnancy in Southeast Asia.

## Methods

### Ethics statement

All study patients provided written informed consent prior to sample collection. The information sheet and consent form were provided in Lao language. If the pregnant woman was unable to discuss consent (e.g., if unconscious or delivering), consent was requested from the patient’s husband or next of kin. If they consented, this was documented through their signatures on the consent form. Ethical approval was granted by National Ethics Committee for Health Research and Oxford Tropical Research Ethics Committee, University of Oxford, UK.

### Study site, patients and clinical procedures

The hospital-based prospective study was conducted in Mahosot Hospital and the Mother and Child Hospital, Vientiane, Laos between February 2006 and November 2010. We recruited all pregnant women admitted with tympanic temperature ≥37.5°C who provided written informed consent. As a pilot study, we planned to recruit 250 pregnant women. Illness history, clinical examination and clinical evolution were recorded on specific case record forms.

Outcomes: the primary outcome was the cause of fever and secondary outcomes were pregnancy outcomes, including: preterm birth (delivery at a gestational age <37 weeks), term but low birth weight (birth weight <2500 g), miscarriage (delivery <28 weeks gestation) [36], stillbirth (delivery >28 weeks gestation with no evidence of life) [38], maternal mortality (death of a pregnant woman or within 42 days of termination of pregnancy, regardless of the duration or the site of pregnancy, from any cause related to or aggravated by the pregnancy or its management) and neonatal mortality (death of a newborn between birth and the first 28 days of life) [39].

### Patient management and follow-up

Women were given acetaminophen (paracetamol) for fever. Women, who presented with signs of a miscarriage or a preterm birth, were transferred to the Gyneco-Obstetrics Ward for management. Antibiotic treatment was based on the results of laboratory tests. After discharge, all pregnant women were asked to return for review and provide convalescence serum on day 10–14 after admission and they were monitored until delivery. If any women presented with a second febrile episode during their pregnancies, the full schedule of examinations and investigations was repeated.

### Laboratory procedures

We focused on specific diseases that local evidence suggested might be important, including septicemia, pyelonephritis, rickettsioses (scrub typhus, murine typhus and spotted fever group rickettsia), leptospirosis, malaria, dengue fever, Japanese encephalitis virus (JEV) infection and HEV infection (S1).

On presentation, all pregnant women had blood (10 mL) drawn for culture [40] and other diagnostic tests discussed below were performed. Ten ml of mid-stream urine sample was taken for culture by inoculation onto Chromogenic agar and incubated at 37°C for 24 hours (Oxoid *Brilliance* UTI Clarity agar, following manufacturer’s instructions).

### Rickettsial pathogens (scrub typhus, murine typhus and spotted fever group rickettsia)

Scrub typhus and murine typhus rapid diagnostic tests (RDTs) were performed for women remaining febrile after 4–7 days of illness as a point-of care testing. AccessBio CareStart Scrub Typhus IgM (CareStart assay, AccessBio) was used to detect anti-*Orientia tsutsugamushi* IgM antibody and GenBio ImmunoDot (ImmunoDot, GenBio, USA) was used for *Rickettsia typhi* anti-IgM antibody.

Subsequently, micro-immunofluorescence assays (IFA) were performed on serum for both scrub typhus (coated with antigen of *O*. *tsutsugamushi* strains Karp, Kato and Gilliam serotypes) and murine typhus coated with *R*. *typhi* Wilmington strain antigens (Australian Rickettsial Reference Laboratory, Geelong Australia). The IFA slides were read on a Nikon ECLIPSE E600 microscope (Nikon Co., Japan). Results were considered positive if IgM or IgG demonstrated a rise in titer of ≥4-fold for paired sera [41].

Rickettsial PCR assays were performed to identify scrub typhus, murine typhus and spotted fever group rickettsia. DNA was extracted from EDTA buffy coat samples with QIAamp DNA Mini kit (QIAGEN AG, Hombrechtikon, Switzerland). The starting sample volume was 200μl and the final elution volume was 100ul. The extracts was divided into 2 aliquots and kept at -80°C. TaqMan real-time PCR assays were used to detect *O*. *tsutsugamushi* (47 kDa *htrA* gene), *Rickettsia* genus (17 kDa gene), and *R*. *typhi* (*ompB* gene) [24].

Rickettsial culture was only performed for patients with positive scrub typhus or murine typhus RDTs. Buffy coat was inoculated onto Vero and L929 cells with inoculation at 35°C in 5% CO_2_ for 6–8 weeks. Identification of rickettsial growth (scrub typhus, murine typhus and spotted fever group rickettsia) was confirmed by IFA and PCR [42].

### Leptospirosis

*Leptospira* spp. culture [43] used the pellet after centrifugation of non anti-coagulated blood and removal of serum, with incubation at room temperature (~25°C) for 12 weeks as described [24]. Microscopic Agglutination Tests (MAT) for *Leptospira* antibodies were performed at the WHO/FAO/OIE collaborating Centre for Reference and Research on Leptospirosis, Queensland Health Forensic and Scientific Services, Australia. A patient was considered to be positive if admission serum showed a titre of 1:400 or if paired sera demonstrated a 4-fold rise [44].

### Dengue

Dengue virus and JEV infections were detected using Panbio ELISAs (PanBio Ltd., Sinnamon Park, Queensland, Australia), i) the JEV-Dengue IgM Combo ELISA (Cat. No. E-JED01C) for the detection of anti-dengue and anti-JEV IgM, ii) Dengue Early ELISA (Cat. No. E-DEN01P) for the detection of dengue NS1 antigen, iii) and Dengue IgG capture ELISA (Cat. No. E-DEN02G) for the detection of high level anti-dengue IgG associated with acute secondary infection. Primary and secondary dengue infections were distinguished using the Dengue IgG indirect ELISA (Cat. No. E-DEN01G) on admission serum when the IgG capture was ELISA negative.

RNA was extracted from sera (140μl) using QIAamp Viral RNA kit (Qiagen) following manufacturer’s instructions in 80μl elution volume. Dengue TaqMan real-time RT-PCR was performed with the SuperScriptIII Platinum One-Step qRT-PCR system (Invitrogen), with 5μl of RNA extract in a 25μl reaction volume [45]. Pan-dengue RT-PCR was performed on all samples and positive samples were submitted to the four specific RT-PCRs for serotyping.

### Hepatitis E Virus infection

HEV ELISA was performed using the Wantai Hepatitis E Virus Diagnostics HEV-IgM ELISA (Cat. No. WE-7196) and HEV-IgG ELISA (Cat. No. WE-7296) (Beijing Wantai Biological Pharmacy Enterprise Co, Beijing, China) according to the manufacturer’s instructions. HEV nested RT-PCR targeting the open reading frame 2 region [46,47] was performed on acute sera for all patients HEV IgM positive on their acute sample.

### Malaria

Malaria smears and Parachek (Pf. HRP-2, Orchid Industries, Goa, India) were performed for pregnant women who came from malaria endemic areas.

Obstetrical ultrasound was performed by sonographers at the Radiology Department, Mahosot Hospital for all pregnant women as soon after admission as possible. The gestational age was estimated by either obstetric ultrasound or last menstrual period.

HIV tests were performed among those with suspected HIV infection (Uni-Gold HIV (Trinity Biotech, Plc) and Determine HIV-1/2 (Inverness Medical Innovations and Abbott Laboratories). Other investigations were requested if clinically indicated.

### Analysis

Data was analyzed using STATA v12 (College Station, Tx). Normally distributed data were shown as means (95% CI) and non-normal distribution as medians (ranges). Categorical variables were shown as frequencies and percentages.

Diagnoses were based on laboratory evidence and on clinical features for two patients with appendicitis (S1). Mixed diseases were defined as patients who had evidence for more than one disease and were classified into 3 grades depending on the strength of the evidence [48] (S2). Severe pregnancy outcome was defined as women who presented with miscarriage, stillbirth, low birth weight, preterm birth, maternal death or neonatal death.

## Results

Between February 2006 and November 2010, 250 pregnant women were recruited. Of these women, 149 (60%) were given a laboratory diagnosis; 132 (53%) had a single disease identified and 17 (7%) had apparent mixed diseases (Table 1). Most (218, 87%) were from Vientiane City, 103 (41%) were unemployed and 123 (50%) were pregnant for the first time. Their median (range) age was 24 (16–45) years and the median (range) admission gestational age was 24 (4–43) weeks. The majority of pregnant women were admitted during the second trimester 111 (45%) and the third trimesters 97 (39%) of pregnancy, and mostly during the rainy season (167, 67%, from May to October).

**Table 1:** Diagnoses of admitted febrile pregnant women from February 2006 to November 2010.

Diseases	Confirmed diagnoses—n (%) (n = 250)
**Single disease**	132 (53)
Dengue	76 (30)
Pyelonephritis	23 (9)
Murine typhus	10 (4)
Scrub typhus	9 (3.6)
*Salmonella* Typhi septicemia (typhoid fever)	6 (2)
Tuberculosis	2 (0.8)
Appendicitis	2 (0.8)
*Staphylococcus aureus* septicemia	1 (0.4)
Leptospirosis	1 (0.4)
Japanese Encephalitis Virus infection	1 (0.4)
*Plasmodium falciparum* malaria	1 (0.4)
**Mixed diseases (grade)**	17 (7)
Dengue + Scrub typhus (II)	4 (2)
Dengue + Murine typhus (II)	4 (2)
Dengue + Pyelonephritis (II (2) & I (2))	4 (2)
Pyelonephritis + Japanese Encephalitis virus infection (II)	1 (0.4)
Scrub typhus + Typhoid fever (II)	1 (0.4)
Dengue + Pyelonephritis + Scrub typhus (I (1) & II (1))	2 (0.8)
Dengue + Pyelonephritis + Murine typhus (II)	1 (0.4)
**No laboratory evidence for pathogens**	101 (40)

The median (range) admission tympanic temperature was 38.5 (37.5–40.5)°C. Fifteen percent of patients stated that they had taken antibiotics before admission (53% of these patients had taken beta-lactam antibiotics such as ampicillin, amoxicillin and penicillin). Headache, myalgia, back pain and arthralgia were described by >60% of patients (Table 2).

**Table 2:** Social-demographic and clinical features of febrile pregnant women by the four most common causes of fevers (single diseases only).

Characteristics	n (%) (n = 250)	Confirmed diagnoses–n (%) (n = 149)	Single disease			
			Dengue fever—n (%) (n = 76)	Pyelonephritis—n (%) (n = 23)	Rickettsioses^#^—n (%) (n = 19)	Typhoid—n (%) (n = 6)
Age*	24 (16–45)	24 (16–45)	24 (17–36)	21 (16–32)	24 (16–35)	24 (19–280)
Ethnic groups+: Lao loum	230 (92)	135 (91)	74 (97)	20 (87)	16 (84)	3 (50)
Lao theung	1 (0.4)	1 (0.7)	-	1 (4)	-	-
Lao soung	14 (5.6)	10 (7)	-	2 (9)	2 (11)	3 (50)
Vietnamese	5 (2)	3 (2)	2 (3)	-	1 (5)	-
Primigravida	123 (50)	76 (51)	39 (51)	16 (70)	9 (47)	1 (17)
Gestational age (weeks) *	24 (4–43)	23 (4–41)	25 (4–41)	21 (8–37)	22 (5–39)	25 (5–38)
Trimester of pregnancy 1^st^	40 (16)	21 (14)	10 (13)	4 (17)	2 (11)	1 (17)
2^nd^	111 (45)	72 (48)	34 (45)	16 (70)	12 (63)	2 (33)
3^rd^	97 (39)	54 (36)	31 (41)	4 (17)	5 (26)	3 (50)
Antibiotic used before admission	36 (15)	18 (12)	7 (9)	2 (9)	4 (21)	2 (33)
Days of fever (days)*	4 (1–120)	4 (1–28)	4 (1–14)	4 (1–28)	7 (1–12)	11 (4–15)
DysuriaDiarrhea	34 (14)27 (11)	21 (14)15 (10)	8 (11)5 (7)	5 (22)0 (0)	2 (11)1 (5)	2 (33)3 (50)
Cough	78 (31)	37 (25)	16 (21)	3 (13)	10 (53)	2 (33)
Bleeding	14 (6)	11 (7)	8 (11)	0 (0)	0 (0)	0 (0)
Temperature on admission (°C)*	38.5 (37.5–40.5)	38.5 (37.5–40.5)	38.5 (37.5–39.5)	38.5 (37.9–40)	38.5 (38–40.5)	39 (38–40)
Rash	14 (6)	12 (8)	10 (13)	0 (0)	1 (5)	0 (0)
Eschar	6 (2)	3 (2)	0 (0)	0 (0)	2 (11)	0 (0)
Abdominal tenderness	13 (5)	7 (5)	0 (0)	2 (9)	0 (0)	0 (0)
Renal angle pain	24 (10)	19 (13)	4 (5)	10 (43)	1 (5)	0 (0)
Lung crepitations	18 (7)	9 (6)	2 (3)	1 (4)	3 (16)	0 (0)
WBC (x10^9^ cells/L)*	9.3 (1.7–36.1)	9 (1.7–36.1)	6.6 (1.7–22.8)	12.8 (5.8–36.1)	9.3 (5.3–14.7)	10.4 (5.6–13.7)
Hematocrit (%)*	32.2 (15.9–49.2)	32 (16.7–49.2)	32.6 (19.8–45.9)	30 (19.3–39)	32.1 (23.2–39)	29 (24–32)
Platelets (x10^9^cells/uL)*	207 (31–735)	193 (31–475)	190 (42–392)	232 (179–376)	200 (108–393)	190 (150–384)
C-Reactive Protein (mg/L)*	39.9 (0.3–210.8)	38.9 (0.4–206.6)	15.3 (0.4–146)	118.5(29–196)	63.9 (24–160)	116 (40–126)
C-Reactive Protein (n = 224) ≥ 10 mg/L [53]	178 (79)	99 (66)	38 (50)	21 (91)	16 (84)	5 (83)

#. Rickettsioses = scrub typhus or murine typhus

*. median (min-max), + ethnic groups are based on IFAD 2012 [66]

Among those (132, 53%) who had a single disease, dengue was the most commonly found (76 (30%)), followed by pyelonephritis (23 (9%)), murine typhus (10 (4%)), scrub typhus (9 (3.6%)) and typhoid fever (6 (2%)). There were also patients with tuberculosis (2 (0.8%)), appendicitis (2 (0.8%)), *S*. *aureus* septicemia (1 (0.4%)), leptospirosis (1 (0.4%)), JEV infection (1 (0.4%)) and *Plasmodium falciparum* malaria (1 (0.4%)) (Table 1). No acute HEV infections were identified, although we found an IgG seroprevalence of 24% indicating prior exposure and possible immunity to HEV.

Of 76 (30%) patients with dengue as a single disease, 71/76 (93%) had positive NS1 and/or PCR assays and, 40/58 (68%) patients had seroconversion; 16/57 (28%) had primary dengue and 41/57 (72%) had secondary dengue. Dengue virus serotype 1 was most commonly detected (56%) (Table 3). Among 76 patients with dengue, 21 (28%) had severe outcome, including 1 maternal death, 6 miscarriages, 2 stillbirths, 9 preterm births, 1 congenital abnormality (ventricular septal defect (VSD)) and 1 vertical transmission (Table 4). Of 76 patients, 28 (37%) had dengue with warning signs and 1 (1%) had severe dengue.

**Table 3:** Laboratory diagnosis results.

Diagnostic tests	Positive patients/total tested patients^#^ (%)
**Conventional blood culture**	
*Escherichia coli*	5/250 (2)^##^
*Salmonella enterica* serovar Typhi	7/250 (3)
*Staphylococcus aureus*	1/250 (0.4)
**Urine culture**	
*Escherichia coli*	28/250 (11)
*Klebsiella oxytoca*	1/250 (0.4)
**Dengue fever**	
**ELISA** (acute sera)	
NS1	62/240 (26)
Seroconverion ^###^	58/179 (32)
**RT-PCR**	71/232 (31)
**Dengue virus serotyping**	
serotype I	40/71 (56)
serotype II	14/71 (20)
serotype III	5/71 (7)
serotype IV	12/71 (17)
**Japanese Encephalitis Virus**	
Seroconversion	1/207 (0.5)
**Hepatitis E (ELISA)**	
Acute (n = 247)*	
**IgM**	4/247 (2)
**IgG**	60/247 (24)
**Rickettsioses**	
Scrub typhus**	
IFA (4-fold rise)	10/111 (9)
PCR	7/217 (3)
Culture	4/22 (18)
2. Murine typhus***	
IFA (4-fold rise)	14/111 (13)
PCR	1/217 (0.5)
Culture	0/22 (0)
**Leptospirosis**	
MAT (4-fold rise)	1/158 (0)
PCR	0/216 (0)

#. Denominators were based on the number of patients who were tested.

##. 3/5 patients also had positive urine culture with *Escherichia coli*.

###. Based on acute and convalescence sera.

* PCR was performed for those who had positive IgM. The results showed negative for all 4 patients.

** 16 patients had evidence of scrub typhus either by IFA (4 fold-rise) or PCR or culture.

*** 15 patients had evidence of murine typhus either by IFA (4 fold-rise) or PCR or culture.

**Table 4:** Pregnancy outcomes for single diseases in pregnant women with fever.

Characteristics	n (%) (n = 228)	Dengue fever (76)—n (%)	Pyelonephritis (23)—n (%)	Murine typhus (10)—n (%)	Scrub typhus (9)—n (%)	Typhoid (6)—n (%)	*S*. *aureus* septicemia (1)—n (%)	TB (2)—n (%)	Leptospirosis (1)—n (%)	JEV (1)—n (%)	Malaria ^4^ (1)—n (%)	Appendicitis (2)—n (%)
Gestational age birth-weeks*	38 (32–44)	39 (33–42)	38 (33–44)	39 (35–40)	38 (35–41)	38 (36–39)	38	-	38	40	-	33 and 34 weeks
Preterm delivery-(<37weeks)	34/186 (18)	9/60 (15)	2/16 (13)	2/7 (29)	1/7 (14)	1/6 (17)	0	-	0	0	-	2/2 (100)
Birth weight-g*	3,000 (1,300–4,200)	3,000 (3,320–3,900)	3,000 (2,300–3,800)	2900 (2500–3500)	3000 (2200–3200)	2500 (1300–3000)	2500	-	2000	3000	-	2199g and 3000g
Term but LBW (<2500g)	11/188 (6)	0/63 (0)	1/16 (6)	0	1/7 (14)	2/5 (40)	0	0	1/1 (100)	0	0	0/2 (0)
Miscarriage	21 (9)	6 (8)	0	0	2 (22)	1 (17)	0	0	0	0	0	0
Stillbirth	5 (2)	2 (3)	1 (4)	0	0	0	0	1 (50)	0	0	0	0
Congenital abnormality	2 (0.9)	1 (1)	0	0	0	0	0	0	0	0	0	0
Vertical transmission	1 (0.4)	1 (1)^1^	-	-	-	-	-	-	-	-	-	-
Maternal death	7 (3)	1 (1)^1^	0	0	0	1 (17)^2^	0	1 (50)^3^	0	0	0	0

*. Median (min-max)

1. Vertical transmission of dengue fever and maternal death: This baby was born from mother who suffered secondary dengue I infection at 39 weeks of gestational age. Baby was born after the mother had 4 days of fever at home. Mother was admitted because of labor. After delivery mother was transferred to ICU because of severe hemorrhage and the baby was also transferred to NICU. Dengue type I was found in mother blood sample and IgM against dengue virus (ELISA) was found in baby’s serum (negative for NS1 and IgG; dengue PCR was not done for the baby because of insufficient blood sample). Mother died 6 days after admitted to ICU but the baby was well until 6 days when the family asked to take the child home.

2. A farmer, 25 year old at 35 weeks of gestational age. She was admitted to hospital because of 4 days of fever, chill, abdominal pain and vomiting. Patient had high temperature on admission (40°C) with bilateral lung crepitations. Ceftriaxone was started but patient deteriorated on day 2 and Cesarean section was performed. Baby was born prematurely (35 weeks) weighing 2000g and was transferred to NICU. Blood cultures from the mother grew *S*. Typhi (susceptible to ampicillin, chloramphenicol, ceftriaxone and ofloxacin) and she died 3 days after admission. The baby was treated with ampicillin and gentamicin; blood cultures were negative. Baby went home well after a week on NICU.

3. A housewife, 20 year old at 32 weeks of gestational age. She was admitted to hospital with 13 days of fever, chill, headache and agitation. No evidence of infection was obtained from blood and urine but CSF showed 29 cmH_2_O of opening pressure, white cells of 750 cells/mm^3^ with 74% lymphocytes and 26% polymorphonuclear leukocytes. CSF glucose and CSF albumin were 2.6mmol/L and 2.2g/L, respectively. Blood glucose was 5.4mmol/L. Gram stain, Indian ink, Zeihl-Neelson and culture were negative. High dose ceftriaxone was started. There was no improvement after one week of treatment and a second lumbar puncture showed no progress with higher white cells (825 cells/mm^3^), low CSF glucose (1.6 mmol/L with blood glucose was 5.6 mmol/L), high protein (1.4 g/L) in CSF and high opening pressure (40 cmH_2_O) but ZN stain for AFB was negative. Sputum ZN stain for TB was negative. 4-drug fixed dose combination (4FDC) was started 2 days after second lumbar puncture (24 days after onset of symptoms). TB was suspected and the patient was started on 4FDC therapy. The patient and family insisted on returning home 2 days after starting 4FDC and the baby did not kick well. The patient did not continue treatment after discharge from hospital and she came back to hospital after a week at home in severely ill and died 10 days after second admission with stillborn baby. The TB culture became positive (fully susceptible) 16 days after she died.

4. A farmer, 28 year old at 26 weeks of gestational age, from Attapeu Province (southern Laos). She was admitted with 6 days of fever, chill, headache and gastric pain. She was diagnosed with malaria (*Plasmodium falciparum*) on the first day of admission to hospital by Parachek-Pf Rapid One Step (malaria blood smears were negative 2 times). She was treated with artemether-lumefantrine for 3 days and returned to Attapeu but was lost to follow-up.

Thirty-one pregnant women were identified as having pyelonephritis. Of these, 26 (84%) patients had positive urine culture only (25 *Escherichia coli* and 1 *Klebsiella oxytoca)*, 2 (6%) had positive blood culture only (*E*. *coli*) and 3 (10%) had both positive urine culture and blood culture (*E*. *coli*). Among pregnant women with pyelonephritis, 23 had a single disease and 8 had mixed-diseases with either dengue, JEV infection, scrub typhus or murine typhus. Low birth weight (1), preterm birth (2) and stillbirth (1) were reported among 23 pregnant women with pyelonephritis only. Of these patients, 97%, 69%, 29% and 20% of *E*. *coli* isolates were susceptible to ceftriaxone, co-amoxicloav, ampicillin and cefalothin, respectively; and 97% received appropriate antibiotics (either ceftriaxone or ampicillin) based on their susceptibilities and drug safety in pregnancy. Of the 30 pyelonephritis patients with *E*. *coli* in urine or blood, one (3%) patient grew an extended spectrum beta-lactamase (ESBL) positive organism.

Evidence for scrub typhus and murine typhus (either IFA with 4 fold-rise in titres, or PCR or culture) were found in 16 (6%) and 15 (6%) of patients, respectively. Of 16 patients with scrub typhus, 8 (50%) were culture and/or PCR positive whilst 8 were diagnosed by only paired sera IFA. In contrast, 14 (93%) of murine typhus patients were diagnosed by paired sera IFA and only one patient was PCR positive (Table 3). Of those patients who had a single disease (10 murine typhus and 9 scrub typhus), 5 (26%) did not receive azithromycin (the anti-rickettsial antibiotic treatment choice at the hospital) or other anti-rickettsial antibiotic, during their admission, as they were diagnosed post-discharge by positive paired sera IFA. Most pregnant women with murine typhus gave birth to healthy babies with normal weight and gestational age, except 2 (20%) who did not received any anti-rickettsial drugs and had preterm births (at 35 and 36 weeks of gestational age). Two pregnant women with scrub typhus had miscarriages and were infected at an early stage of pregnancy (one woman at 5 weeks and another at 15 weeks). Both came to hospital after two weeks of illness, were given azithromycin and although the mothers recovered, they had fetal loss.

Seven pregnant women had *Salmonella enterica* serovar Typhi septicaemia. Six women had a single disease, whereas one had apparent mixed diseases (Grade II) with scrub typhus. Five of them had poor outcomes, including maternal death (1), miscarriage (1), term-low birth weight (2) and preterm birth (1). All *S*. Typhi isolates were susceptible to ampicillin, ceftriaxone, ofloxacin and chloramphenicol. All pregnant women with positive *S*. Typhi blood cultures received ceftriaxone during their admission.

Of 149 women with confirmed diagnoses, 38/135 (28%) had severe pregnancy outcomes. Of those with single disease with severe outcomes, 28 (78%) had dengue fever, scrub typhus, murine typhus or typhoid fever. There was no significant difference in frequency of severe outcome between those who had single disease and mixed diseases (p = 0.35). Of 17 (7%) patients with mixed diseases, 3 (18%) had grade I and 14 (82%) had grade II evidence. Apparent mixed diseases were mainly found among those who had dengue and rickettsioses (2 patients were rickettsial PCR positive and 10 patients had positive paired sera IFA) (Table 2). There were no significant differences in the proportion of women who had severe pregnancy outcome between those with grade I and grade II mixed diseases (p = 0.40).

Median (range) high-sensitivity-C-Reactive Protein (CRP) was higher among those who had bacterial diseases (pyelonephritis, scrub typhus and murine typhus, typhoid, leptospirosis, tuberculosis and *S*. *aureus* septicemia) than those who had viral diseases (dengue and JEV infection) at 63.5 (0.3–210.8) mg/L and 15.5 (0.4–146) mg/L, respectively (p<0.0001). Of 250 pregnant women, 146 (59%) received antibiotics during admission. Based on 10mg/L as high sensitivity-CRP cut-off point [49], there was strong association (Chi-square test) between antibiotics used in hospital and high CRP (p<0.0001). If CRP > 10 mg/L was used as the only diagnostic criteria to determine whether a patient received antibiotics 5% of pregnant women who should have received one would not have received an antibiotic. Twenty-six percent of pregnant women with a CRP < 10 mg/L received antibiotics.

Of 250 pregnant women, 101 (40%) did not have confirmed diagnoses. Forty-four patients (18%) came to hospital with influenza-like illnesses [50] and 22/44 (50%) of them did not have laboratory confirmation diagnoses. HIV infection was tested for 7 (3%) patients and 4/7 (57%) were positive.

## Discussion

Dengue was the most common aetiology found during 57 months of monitoring pregnant women admitted with fever at two central hospitals in Vientiane. Other common causes included pyelonephritis, scrub typhus, murine typhus and typhoid. A similar study of pregnant women on the Thailand-Myanmar border, 646 km to the southwest, showed that malaria was the most common cause of fever during pregnancy, followed by pyelonephritis, rickettsioses and dengue fever [36]. Different study areas and periods will result in a different pattern of aetiology of fever but, apart from malaria, this study and that of McGready *et al*. [36] gave similar results. Although malaria has been a common cause of infection on the Thailand-Myanmar border [51], there is thought to be no malaria transmission in Vientiane. On the Thailand-Myanmar border arthropod-borne (malaria, rickettsioses and dengue) and zoonotic disease (leptospirosis) accounted for 47% of all febrile illnesses in pregnancy [36]; in Vientiane they accounted for 46%, supporting the importance of these pathogens in pregnancy in Southeast Asia.

In Vientiane, severe pregnancy outcomes were found in mothers with dengue. A systematic review of dengue fever during pregnancy showed a similar pattern of poor pregnancy outcomes [29]. Although congenital abnormalities have rarely been reported, Sharma *et al*. (1992) reported neural tube defects among children born from mothers with dengue, but dengue diagnosis was based on clinical features only [52]. Congenital heart defects from maternal hyperthermia in the first trimester of pregnancy have been described in many studies [14], leading to uncertainty as to the importance of individual pathogens, rather than fever *per se*. The importance of dengue as a pathogen in pregnancy will presumably vary with the great inter-year differences in dengue incidence in the general population. Further prospective investigation as to whether pregnancy increases the risk of dengue and disease severity is needed [53,54]. Given the fluid balance changes in both pregnancy and dengue, an interaction is plausible and fluid management protocols in pregnancy may need to be adapted from those used for non-pregnant adults [55].

Pyelonephritis was the second most frequent cause, as was found by McGready *et al*. on the Thailand-Myanmar border, where pyelonephritis was the most common cause of fever in pregnancy after malaria [36]. Although only one patient with ESBL was described here, the frequency of these multi-drug resistance pathogens has increased in Laos [56] and appropriate susceptibility-profile guided antibiotics will be vital for treating individual patients and for antibiotic stewardship in communities.

We also found that scrub typhus was associated with severe pregnancy outcomes, especially when pregnant women were infected at an early stage of pregnancy [30,36]. Murine typhus was less frequently described in pregnancy than scrub typhus. However, we found adverse pregnancy outcomes with murine typhus, as has been described elsewhere [36]. In this study, 93% of murine patients were diagnosed by paired sera IFA whilst only 7% were PCR positive. We are not aware of evidence on the duration of *Rickettsia typhi* bacteraemia. Our PCR positive *R*. *typhi* patient had 8 days of fever before admission to hospital. Wattanaworawit *et al*. [57] found among positive paired sera *R*. *typhi* IFA patients, 36% were also diagnosed by PCR. Murai *et al*. [58] found that *Orientia tsutsugamushi* DNA was detectable in patient’s blood until day 3–8 after the start of chemotherapy with minocycline or doxycycline. Scrub typhus and murine typhus are known as a major cause of rickettsial diseases in Southeast Asia [59,60]. Few cases of spotted fever group rickettsia have been reported in Lao patients [59,61] and we did not find any evidence for these pathogens in the Lao pregnant febrile women described here.

We categorized apparent mixed infections based on a high likelihood grade of diagnosis (S1 and S2) [48]. Patients with mixed diseases mainly had dengue (60% of positive PCR/Antigen detection, 40% of seroconversion) and rickettsioses (~83% positive paired sera IFA and 17% of positive PCR). Mixed diseases were also described in previous studies both in pregnant and non-pregnant women [36,62], but caution should be used when mixed diseases are based just on serology [48].

Important limitations of this study include that it was hospital, rather than community, based and therefore could not estimate the incidence of causes of fevers amongst pregnant women in the community and we were not able to recruit matched afebrile pregnant women. Hence, we were unable to investigate the frequency of asymptomatic carriage of the pathogens examined. Forty percent of pregnant women in this study did not have confirmed diagnoses; of these 18% received antibiotics before collecting samples for cultures, which may have reduced the frequency of positive culture results [63]. We focused on key pathogens describing in previous studies in this area [24,36,40]. We were not able to investigate diverse other important viral infections in pregnancy [64] or other potential pathogens such as *Coxiella burnetii*. In addition, convalescence samples were not available for all serological investigations (17%, 37% and 56% without convalescence samples for dengue/JEV ELISA, leptospiral MAT and scrub/murine typhus IFA, respectively) and we may therefore have missed some patients who seroconverted. Approximately 20% of patients came to hospital with influenza-like illnesses but we were unable to test for respiratory viruses. Few patients were suspected with HIV infection. At the time of the study universal HIV testing in pregnant women was not routine practice in Laos and needs to be expanded. By 2015, 50% of Lao pregnant women are expected to receive counseling and HIV testing [65].

Large community based cohort studies, with extended diagnostic testing, would help us to deal with these limitations. However, data from this study provides evidence of the cause of febrile illnesses in pregnant women admitted to hospital and will guide antimicrobial prescribing in this vulnerable group where laboratory facilities are limited.

These data suggest that dengue control, optimization of dengue-related fluid balance management during pregnancy and optimization of early diagnosis and treatment of rickettsial diseases and typhoid in pregnancy are needed.

## Supporting Information

S1 Appendix Definitions of confirmed diagnoses. (DOCX)

S2 Appendix Grades of Mixed diseases. (DOCX)
