# Supplementary material for: The Aetiologies and Impact of Fever in Pregnant Inpatients in Vientiane, Laos
Source: PLoS Negl Trop Dis. 2016 Apr 6;10(4):e0004577. doi: 10.1371/journal.pntd.0004577 (PMC4822858; doi:10.1371/journal.pntd.0004577)
Supplement: S1 Appendix — (DOCX) [file pntd.0004577.s001.docx]

**S1. Definitions of confirmed diagnoses.**

Confirmed diagnosis was based on culture (ie, blood, rickettsial, and leptospiral cultures), antigen/protein detection, serology testing for Dengue/JEV ELISA (seroconversion) and 4 fold-rise in titre of microscopic agglutination tests and immunofluorescence assay (IFA).

| Diseases | Diagnostic tools |
| --- | --- |
| Septicemia | Evidence of clinically significant bacterial pathogen in blood (positive blood culture) |
| Pyelonephritis | Systemic symptoms such as fever, chills, nausea, vomiting, flank pain were accompanied by a positive urine culture, and/or positive blood culture |
| Rickettsioses  1. Scrub typhus and murine typhus | Evidence of 4 fold-rise in titres of IgM or IgG by Immunofluorescence assay (IFA) or positive PCR or positive culture |
| 2. Spotted fever group | Positive PCR or positive culture |
| Leptospirosis | Evidence of 4 fold-rise in titres by microscopic agglutination tests (MAT) or positive PCR or positive culture |
| Malaria | Positive malaria Ag HRP2 and/or positive malaria blood smear |
| Dengue fever | Evidence of seroconversion by ELISA, or detection of dengue NS1 protein or positive dengue RT-PCR using serum |
| Japanese Encephalitis | Evidence of seroconversion by anti-JEV IgM ELISA in serum |
| Hepatitis E | Evidence of seroconversion by ELISA or positive PCR |
