# Supplementary material for: The Aetiologies and Impact of Fever in Pregnant Inpatients in Vientiane, Laos
Source: PLoS Negl Trop Dis. 2016 Apr 6;10(4):e0004577. doi: 10.1371/journal.pntd.0004577 (PMC4822858; doi:10.1371/journal.pntd.0004577)
Supplement: S2 Appendix — (DOCX) [file pntd.0004577.s002.docx]

**S2. Grades of Mixed diseases.**

| Grades | Definitions |
| --- | --- |
| Grade I | Culture or molecular detection of both pathogens or direct observation such as in a malaria film |
| Grade II | Serological diagnosis with either seroconversion or fourfold antibody responses to both pathogens, without evidence of cross-reactions, or using Western blotting |
| Grade III | Serological diagnosis based on admission serology without exclusion of cross reactions or antibody persistence or culture, molecular, or admission serological detection |
